# Prevalence and molecular characterization of *Cryptosporidium* spp. and *Giardia duodenalis* in humans and dogs from Fujian Province, Southeast China

**DOI:** 10.1016/j.fawpar.2025.e00278

**Published:** 2025-07-23

**Authors:** Si-Ang Li, Yu-Ling Lin, Yun-Peng Bai, Fuli Wen, Li-Yuan Huang, Wen-Yuan Miao, Dong-Hui Zhou

**Affiliations:** a Key Laboratory of Fujian-Taiwan Animal Pathogen Biology, College of Animal Sciences, Fujian Agriculture and Forestry University, Fuzhou 350002, China; b Key Laboratory of Animal Pathogen Infection and Immunology of Fujian Province, College of Animal Sciences, Fujian Agriculture and Forestry University, Fuzhou 350002, China; c Shengli Clinical Medical College of Fujian Medical University, Fujian Provincial Hospital, Fuzhou University Affiliated Provincial Hospital, Fuzhou 350001, China

**Keywords:** *Cryptosporidium*, *Giardia duodenalis*, Dog, Human, Zoonotic infection, Fujian Province

## Abstract

*Cryptosporidium* spp. and *Giardia duodenalis* are two well-known protist pathogens which can result in diarrhea in humans and animals. The two parasites have been detected in humans and dogs worldwide with diverse species and genotypes of various levels and zoonotic potential and public health concern. This investigation aimed to determine the prevalence and molecular characteristics of *Cryptosporidium* spp. and *G. duodenalis* in humans and dogs in Fujian province, China. A total of 1149 fecal samples (643 from humans and 506 from dogs) were collected from nine districts in Fujian Province. Detection of *Cryptosporidium* was performed using nested PCR targeting the *SSU rRNA* gene, while *G. duodenalis* was detected by amplification three genes including the beta-giardin, glutamate dehydrogenase, and triosephosphate isomerase. No *Cryptosporidium* or *G. duodenalis* were detected in any of the human samples tested. In contrast, the prevalence of *Cryptosporidium* in dog samples was found to be 1.2 % (6/506), while the infection rate of *G. duodenalis* was detected in 0.4 % (2/506) of the dog samples. According to the age analysis, all samples infected with *Cryptosporidium* 2.2 % (6/268) and *G. duodenalis* 0.8 % (2/268) were from dogs ≤1 year. Sex-based analysis indicated that the infection rate of *Cryptosporidium* was slightly higher in male dogs (1.2 %, 3/248) compared to female dogs (1.2 %, 3/258). Additionally, *G. duodenalis* was detected in 0.8 % (2/248) of male dogs, while no positive samples were observed in female dogs. Phylogenetic analyses further identified *C. canis*, a zoonotic species of *Cryptosporidium*, as well as two zoonotic assemblages (C and D) of *G. duodenalis*. These results provide preliminary reference data for monitoring *Cryptosporidium* and *G. duodenalis* infections in both humans and dogs, and also offer essential support for future prospective studies.

## Highlights

• All 643 human samples negative for *Cryptosporidium* spp. and *G. duodenalis* infections.
• *Cryptosporidium* (1.19 %, 6/506) and *G. duodenalis* (0.40 %, 2/506) identified in dogs.
• Higher prevalence of infection among young (≤1 year) and male dogs.
• Presence of *C. canis* and *G. duodenalis* assemblages C and D in dogs suggests potential zoonotic risk.

## 1 Introduction

Diarrhea is a global issue, and it was the eighth leading cause of death across all age groups in 2016 (Collaborators, 2018). Diarrhea is the leading cause of death in young children, and even in developed countries, acute diarrhea can develop into gastrointestinal emergencies, leading to disease deterioration (Disease et al., 2016). Bacteria, viruses, and parasites are all important pathogens that cause diarrhea. Among the latter, *Cryptosporidium* spp. and *Giardia duodenalis* are common diarrheal pathogens in humans and animals worldwide (Naguib et al., 2018; Checkley et al., 2015). *Cryptosporidium* and *G. duodenalis* are one of the most prevalent intestinal parasites worldwide, with high prevalence in both developing and developed countries (Einarsson et al., 2016; Mahmoudi et al., 2017; Cai et al., 2021). *Cryptosporidium* spp. and *G. duodenalis* are transmitted to humans via the fecal-oral route, either directly through person-to-person contact or contact with infected animals, or indirectly through the consumption of contaminated food or water, leading to foodborne or waterborne transmission (Ryan and Caccio, 2013; Checkley et al., 2015).

*Cryptosporidium* spp. and *G. duodenalis* are significant intestinal protozoa and have become a primary focus of global public health concerns due to their widespread impact on human health (Fakhri et al., 2021). To date, 40 species and over 70 genotypes of *Cryptosporidium* spp. have been identified, among which six species, including *C. hominis*, *C. parvum*, *C. meleagridis*, *C. cuniculus*, *C. felis*, and *C. canis*, are more commonly associated with human infections (Chalmers and Katzer, 2013; Li et al., 2024). In comparison, nine species of *Giardia.* spp. has been identified such as *G. duodenalis*, *G. microti*, *G. muris*, *G. agilis*, *G. ardeae*, *G. psittaci*, *G. varani*, *G. peramelis*, and *G. crispidarum*. They infect a wide range of animals including birds, amphibians, rodents and mammals (Ryan et al., 2021; Li et al., 2023; Li et al., 2017). Among these, *G. duodenalis* (also known as *G. lamblia* or *G. intestinalis*) is the specie most frequently found in humans, as well as in various livestock, wild animals, and companion animals (Einarsson et al., 2016). *G. duodenalis* is known as a multispecies complex (Li et al., 2023), with a total of eight genetically distinct assemblages (A–H) (Wu et al., 2020). Each assemblage exhibits distinct host specificity and genetic diversity. Assemblages A and B are zoonotic and have been reported in both mammals and humans (De Aquino et al., 2020). Additionally, assemblages C and D are primarily found in canids, while assemblage E is prevalent in ruminants and livestock; assemblage F is identified in cats, whereas assemblages G and H are associated with rodents and marine animals (seals) (Li et al., 2023; Ryan and Zahedi, 2019; Mahmoudi et al., 2017). Although assemblages A and B are the primary zoonotic types, recent studies have reported the presence of assemblages C, D, E, F in humans (Sprong et al., 2009; Sui et al., 2022; Pipikova et al., 2020), suggesting potential zoonotic spillovers or host adaptations. This suggests that close contact between humans and animals may facilitate the transmission of a variety of intestinal parasites, increasing the risk of cross-species infections.

In China, *Cryptosporidium* spp. and *G. duodenalis* have been detected in a wide range of hosts, including carnivores, lagomorphs, primates, birds, and rodents (Li et al., 2023; Yang et al., 2018). In recent years, companion animals such as dogs and cats have become an integral part of society. Approximately 17 % of Chinese households now own at least one companion animal, with dogs being the most popular choice (Sui et al., 2022). As the number of companion animals continues to grow and the potential risk of zoonotic disease transmission increases, there has been a rise in people's awareness of preventing and controlling zoonotic parasitic diseases. The close relationship between humans and companion animals, particularly in urban environments, facilitates the spread of zoonotic pathogens, including *Cryptosporidium* spp. and *G. duodenalis*, but detailed data on the prevalence of *Cryptosporidium* spp. and *G. duodenalis* in pet dogs in Fujian Province remain limited. Dogs are known to play an important role in the transmission of these two pathogens, which makes it crucial to understand their potential as zoonotic reservoirs. Thus, we conducted an investigation to determine the prevalence of *Cryptosporidium* spp. and *G. duodenalis* in humans and dogs in Fujian Province and to evaluate their potential role in the zoonotic transmission of human cryptosporidiosis and giardiasis.

## 2 Materials and methods

### 2.1 Participant recruitment

In this study, human subjects were recruited from regional hospitals across multiple districts, consisting of randomly selected outpatients. Only subjects who provided informed consent were enrolled, with a single sample collected from each participant. Animal samples were collected from pet hospitals, pet shops, animal shelters, and breeding kennels. These places were selected as they serve as primary activity areas for pet owners and key sources of pets. Written informed consent was obtained from all participating owners.

### 2.2 Study areas and sample collection

**Fig. 1:**
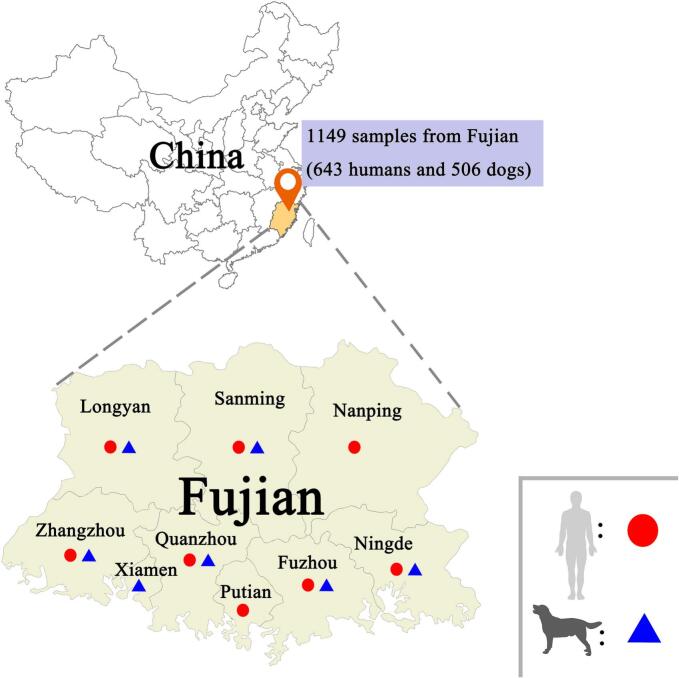
Distribution of sampling sites in Fujian. ●: human fecal samples, ▲: dog fecal samples.

Fujian province is located to the southeast of China and consists of six coastal cities and three inland cities (Fu et al., 2020). The total land area of Fujian covers 124,000 km^2^, with a marine area of 136,000 km^2^. Characterized by lofty peaks and ranges, continuous hills interspersed with river valleys and basins, mountainous and hilly terrains account for over 80 % of the provinces total area (Ke et al., 2013). From September 2020 to February 2022, a total of 1149 fecal samples were collected from nine areas in Fujian Province (Longyan, Fuzhou, Quanzhou, Zhangzhou, Putian, Xiamen, Sanming, Nanping and Ningde), including 643 human fecal samples and 506 dog fecal samples (Fig. 1, Table 1, Table 2). The human fecal sample included 357 males and 286 females. These human fecal samples were further categorized into 461 normal samples and 182 diarrheal samples. The age categorization consisted of 143 samples from ≤13 years, 238 from 13 to 17 years, and 262 from ≥18 years. Canine fecal samples were collected from various sources across 7 districts including 257 samples from 36 pet hospitals, 116 from 33 pet shops, 91 from 4 stray animal shelters, and 42 from 1 breeding kennels. Among the dog samples, the number of male and female samples was 248 and 258 respectively. These samples included 477 normal fecal samples and 29 diarrheal samples. The age distribution of dog samples included 268 from dogs aged ≤1 year and 238 from dogs older than 1 year. The collected samples were placed in sterile collection tubes, stored on ice, and then transferred to the laboratory. Each sample was labeled with the date of collection and its source. Fresh fecal samples were stored at −80 °C in an ultra-low temperature freezer immediately after collection.

**Table 1:** Sampling information of human fecal samples in Fujian province.

Areas	Gender		Age			Diarrheal	Summation
			≤13	13–17	≥18		
Longyan	Male	40	11	8	21	16	58
	Female	18	8	10	0	9	
Fuzhou	Male	69	15	24	30	8	109
	Female	40	16	8	16	11	
Quanzhou	Male	48	18	20	10	12	101
	Female	53	22	16	15	18	
Zhangzhou	Male	35	7	9	19	10	59
	Female	24		14	10	5	
Putian	Male	51	10	15	26	21	76
	Female	25	3	6	16	0	
Xiamen	Male						
	Female						
Sanming	Male	36	7	22	7	19	64
	Female	28	11	14	3	8	
Naping	Male	27	2	17	8	6	78
	Female	51	10	21	20	17	
Ningde	Male	51	3	18	30	5	98
	Female	47		16	31	17	
Total		643	143	238	262	182	643

**Table 2:** Sampling information of dog fecal samples in Fujian province.

Areas	Gender		Age		Sources				Diarrheal	Summation	Prevalences % (NO.) (95 % CI)			
			≤1	>1	Pet hospitals	Pet shops	Animal shelters	Breeding kennels			*Cryptosporidium*	*p*-Value	*Giardia duodenalis*	*p*-Value
Longyan	Male	31	20	11	5	17	3	6	6	65			1.5 % (1) (0–4.6)	0.481
	Female	34	16	18	7	18	9							
Fuzhou	Male	65	32	33	36	3	16	10		137	0.7 % (1) (0–2.2)	0.628	0.7 % (1) (0–2.2)	0.628
	Female	72	22	50	54	9	9							
Quanzhou	Male	59	30	29	29	14	8	8		106	1.9 % (2) (0–4.5)	0.434		
	Female	47	19	28	36	6	5		6					
Zhangzhou	Male	8	3	5	1	2	0	5	1	32	Reference		Reference	
	Female	24	18	6	8	8	8							
Xiamen	Male	16	5	11	1	10	1	4	4	44	4.6 % (2) (0–11.0)	0.222		
	Female	28	21	7	3	6	16	3						
Sanming	Male	41	28	13	30	11			8	76	1.3 % (1) (0–3.9)	0.514		
	Female	35	27	8	29		6							
Ningde	Male	28	23	5	11	9	8		4	46				
	Female	18	4	14	7	3	2	6						
Total		506	268	238	257	116	91	42	29	506				

### 2.3 Genomic DNA extraction

Genomic DNA of all fecal samples were extracted using the E.Z.N.A.® fecal DNA kit (OMEGA, CT, USA). The extracted DNA was placed in a − 80 °C refrigerator until use.

### 2.4 PCR amplifications

The nested-PCR method was used to *Cryptosporidium* spp. detection by targeting an 840 bp fragment of the small subunit (*SSU*) rRNA gene (Xiao et al., 1999). Additionally, nested-PCR was employed to amplify the beta-giardin (*bg*), glutamate-dehydrogenase (*gdh*), and triosephosphate isomerase (*tpi*) genes of *G. duodenalis* (Caccio et al., 2008; Lalle et al., 2005; Sulaiman et al., 2003). The size of the gene fragment for *bg*, *gdh*, and *tpi* is 511 bp, 530 bp and 530 bp respectively. In all PCR assays, sterile ultrapure water was used instead of template DNA as a negative control, while the DNA of *C. parvum* and *G. duodenalis* (stored at −80 °C in the lab) served as positive controls, respectively. PCR reactions were carried out using a thermocycler, with 2 μL of genomic DNA (for primary PCR) or 2 μL of PCR amplification product (for secondary PCR). The PCR reaction system (25 μL for each sample) was as follows: 10 × PCR Buffer, 2.5 μL; dNTPs (25 mM), 2 μL; Ex-Taq polymerase, 0.2 μL; each primer (10 μM), 0.25 μL; MgCl_2_, 1.5 μL; and deionized water, 16.3 μL (all form Takara Bio, Dalian, China). Each PCR amplification product was subsequently examined by electrophoresis in a 2.0 % agarose gel containing 1 μg/mL of ethidium bromide. All positive secondary PCR products were sent to Sangon Biotech Company (Shanghai, China) for bidirectional sequencing. Primers of the secondary PCR were used for sequencing in both forward and reverse directions.

### 2.5 Sequence and phylogenetic analysis

After sequencing, the results were spliced using SeqMan Pro software (DNASTAR, Inc.). The spliced sequences were then compared with NCBI reference sequences and analyzed using MegAlign (DNASTAR, Inc.) to identify *Cryptosporidium* spp. and *G. duodenalis*. Then, the phylogenetic trees were constructed using the MEGA11 program by the maximum-likelihood (ML) method with 1000 bootstrap replicates (https://www.megasoftware.net/).

### 2.6 Statistical analysis

Variations in the occurrence of *Cryptosporidium* spp. and *G. duodenalis* in human and dog according to age, sex, diarrhea situations, and samples source were analyzed by X^2^ test using SPSS 20.0 (SPSS 20.0, IBM, USA). Each of these variables was included in the binary logit model as an independent variable by multivariable regression analysis. When the *P* < 0.05, the results were considered statistically significant.

## 3 Results

In this study, a total of 1149 fecal samples (643 human samples from eight areas and 506 dog samples from seven areas) were collected in nine areas from Fujian Province, China. These samples were subjected to DNA testing using nested-PCR, targeting the *Cryptosporidium* spp. *SSU rRNA* gene and the *G. duodenalis bg*, *gdh* and *tpi* genes respectively. The results showed that no *Cryptosporidium* spp. and *G. duodenalis* were detected in the 643 human fecal samples (461 normal and 182 diarrheal), resulting in a prevalence rate of 0 % (0/643). In contrast, a total infection rate of *Cryptosporidium* and *G. duodenalis* in dogs was 1.2 % (6/506) and 0.4 % (2/506) respectively (Table 2). The analysis of *Cryptosporidium* prevalence in dog samples from different regions showed that Xiamen city had the highest at 4.6 % (2/44). In comparison, the infection rates in Fuzhou, Quanzhou, and Sanming were lower, at 0.7 % (1/137), 1.9 % (2/106), and 1.32 % (1/76), respectively (Table 2). Notably, all samples from Longyan, Ningde, and Zhangzhou tested was negative for *Cryptosporidium*. However, the prevalence surveys of *G. duodenalis* in dogs revealed that the infection rate was 0.7 % (1/137) and 1.5 % (1/65) in the Fuzhou and Longyan areas, respectively. Statistical analysis indicated that there was no significant difference in the prevalence of *Cryptosporidium* and *G. duodenalis* across different regions (*P* > 0.05).

### 3.1 Impact of different factors on *Cryptosporidium* and *G. duodenalis* infections

The analysis of *Cryptosporidium* and *G. duodenalis* infections in dogs based on sample sources revealed varying prevalence rates. The prevalence of *Cryptosporidium* infection was 1.2 % (3/257) in samples collected from pet hospitals, 2.2 % (2/91) in animal shelters, and 2.4 % (1/42) in breeding kennels (Table 3). In contrast, *G. duodenalis* was detected only in samples from pet hospitals, with a prevalence rate of 0.9 % (2/257). Age-based analysis showed that the prevalence of *Cryptosporidium* infection was highest in dogs aged ≤1 year, at 2.3 % (6/268) (χ^2^ = 5.392, *p* < 0.05) (Table 3). The prevalence of *G. duodenalis* in dogs ≤1 year old was 0.8 % (2/268) (χ^2^ = 1.783, *P* > 0.05), indicating no significant association between age and *G. duodenalis* infection. Sex-based analysis revealed that the prevalence of *Cryptosporidium* infection was 1.2 % (3/248) in male dogs and 1.2 % (3/258) in female dogs, which was not statistically significant (χ^2^ = 0.002, *P* > 0.05) (Table 3). *G. duodenalis* was detected in only 0.8 % (2/248) of male dogs (χ^2^ = 2.089, *P* > 0.05). When comparing fecal samples of diarrheal and normal dogs, *Cryptosporidium* was found in 3.4 % (1/29) of diarrheal samples and 1.1 % (5/477) of normal samples (χ^2^ = 1.344, *P* > 0.05), with no significant difference. However, *G. duodenalis* was detected in 6.9 % (2/29) of the diarrheal samples, a statistically significant difference when compared to normal samples (χ^2^ = 33.027, *P* < 0.05) (Table 3). Analysis of dogs samples from different sources showed that the infection rate of *Cryptosporidium* in pet hospital samples was 1.17 % (3/116) (χ^2^ = 2.388, *P* > 0.05), 2.20 % (2/42) in animal shelter samples (χ^2^ = 4.399, *P* < 0.05), and 2.38 % (1/24) in breeding kennel samples (χ^2^ = 3.825, *P* < 0.05). *G. duodenalis* was only detected in samples from pet hospitals, with an infection rate of 0.78 % (2/116) (χ^2^ = 1.584, *P* > 0.05). These results indicate that there are statistically significant differences between different sample sources. These findings highlight the importance of monitoring and controlling *Cryptosporidium* and *G. duodenalis* infections in various dog populations.

**Table 3:** Analysis of infection rates of *Cryptosporidium* and *Giardia duodenalis* in dogs, classified by age, gender, diarrhea status and source.

Type		Number	Prevalences % (NO.) (95 % CI)			
			*Cryptosporidium*	*p*-Value	*Giardia duodenalis*	*p*-Value
Age	≤1	268	2.2 % (6) (0.5–4.0)	0.020	0.8 % (2) (0–1.8)	0.182
	>1	238				
Gender	Male	248	1.2 % (3) (0–2.6)	0.961	0.8 % (2) (0–1.9)	0.685
	Female	258	1.2 % (3) (0–2.5)	0.961		
Health	Normal	477	1.1 % (5) (0.1–2.0)	0.246		
	Diarrheal	29	3.4 % (1) (0–10.5)	0.246	6.9 % (2) (0–16.7)	0.009
Sources	Pet hospitals	116	1.2 % (3) (0–5.5)	0.122	0.8 % (2) (0–4.1)	0.208
	Pet shops	91		Reference		Reference
	Animal shelters	42	2.2 % (2) (0–11.5)	0.036		
	Breeding kennels	24	2.4 % (1) (0–12.8)	0.050		

### 3.2 Cryptosporidium species

**Fig. 2:**
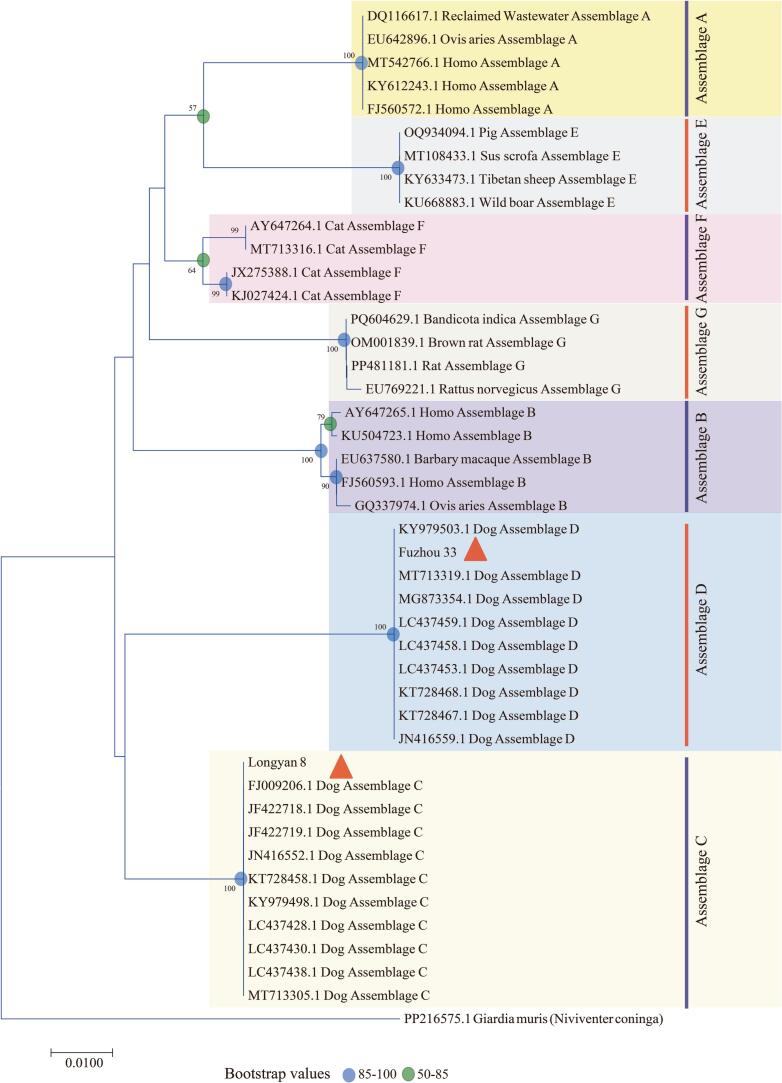
The phylogenetic tree illustrating the evolutionary relationships of *Giardia duodenalis* from dog feces was constructed based on bg gene sequences using maximum likelihood analysis. The triangle indicates the positive samples in this study.

Six *Cryptosporidium* detected positive samples were analyzed using SSU rRNA gene sequence, and only *C. canis* was identified. Three of the *Cryptosporidium* isolates showed 100 % similarity to the canine *Cryptosporidium* reference sequences available in the GenBank database (*C. canis*, GenBank accession numbers KT749818, KF516543, and KF516543). The remaining *Cryptosporidium* isolates showed 100 % similarity to the reference sequence of a human *Cryptosporidium* isolate (*C. canis*, GenBank accession number KT749817). Phylogenetic relationship analyses were conducted using MEGA 11 software, employing Clustal W for sequence alignment, and the identity of *Cryptosporidium* species was confirmed via maximum likelihood (ML) analysis (Fig. 2).

### 3.3 *G. duodenalis* genotype

**Fig. 3:**
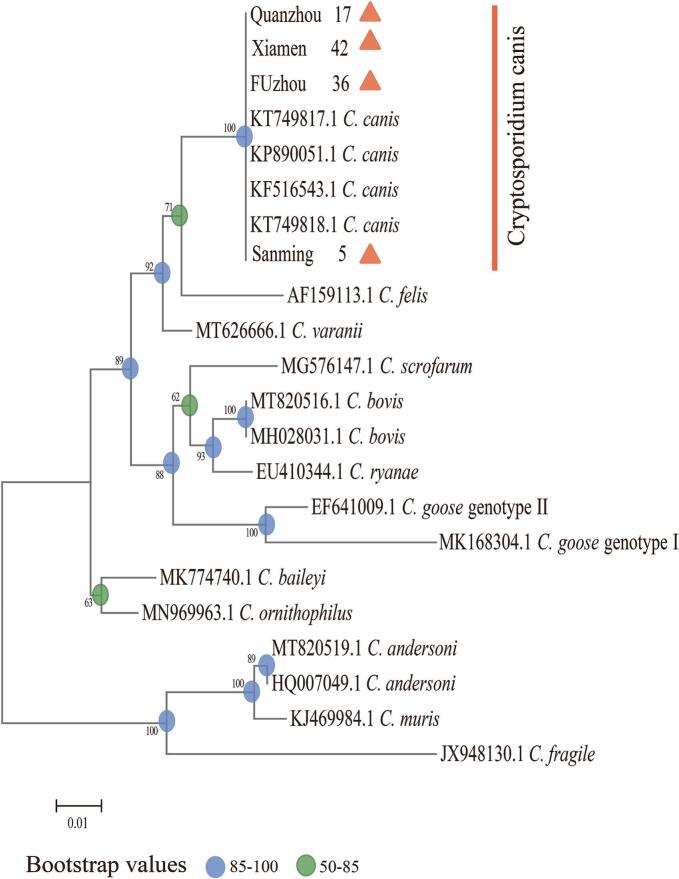
The phylogenetic tree illustrating the evolutionary relationships of *Cryptosporidium* from dog feces was constructed based on *SSU rRNA* gene sequences using maximum likelihood analysis. The triangle indicates the positive samples in this experiment.

PCR amplification targeting three gene loci (*bg*, *gdh*, and *tpi*) of *G. duodenalis* revealed that successful amplification and sequencing were only achieved at the *bg* loci, while the amplification of the *gdh* and *tpi* loci consistently failed despite repeated molecular detection using various primers. DNA sequencing and subsequent analysis of the PCR products based on the *bg* loci from two *G. duodenalis*-positive specimens revealed the presence of two known *G. duodenalis* genotypes (C and D). Genotype C showed 100 % homology to sequence MT713305 (from American dogs), while genotype D exhibited 100 % homology to sequence KY979503 (from Chinese dogs) (Fig. 3).

## 4 Discussion

Due to the rapid development of the global economy, per capita living standards have increased, and overall quality of life has improved. Although diarrhea-related deaths have decreased in recent decades, hundreds of thousands of children still die from diarrhea each year (Liu et al., 2016). Diarrheal diseases remain a serious health problem in many parts of the world, especially in resource-poor settings (Mortality and Causes of Death, 2015; Liu et al., 2012). Poor sanitation is a major cause of diseases by fecal-oral transmission, including diarrhea which is responsible for more than 1.2 million deaths annually (Majorin et al., 2017). Some human intestinal diseases can be attributed to contact with food animals and pets (Hale et al., 2012; Conrad et al., 2017), and waterborne and environmental transmission are also significant factors (Naguib et al., 2018). In recent years, companion animals such as dogs and cats have become an integral part of society. Approximately 17 % of Chinese households now own at least one companion animal, with dogs being the most popular choice (Sui et al., 2022). A white paper on China pet industry showed that the number of pet dogs in Chinas cities and towns was 55.03 million, and the number of pet cats was 44.12 million by 2019 (Zhou et al., 2020). The market economic generated by pets and pet owners reached 202.4 billion yuan. The increase in pet ownership highlights the importance of implementing effective hygiene and disease detection measures to mitigate the transmission of enteric diseases from animals to humans.

In this study, we analyzed human (eight areas) and dog (seven areas) fecal samples collected from nine districts in Fujian Province to assess the prevalence of *Cryptosporidium* and *G. duodenalis* infection. Notably, neither *Cryptosporidium* nor *G. duodenalis* were detected in any of the human fecal samples tested. A total of 643 human fecal samples were obtained from regional hospitals in Fujian Province, comprising 461 samples of normal feces and 182 samples of diarrheal feces. Among these, 143 fecal samples were from individuals ≤13 years, with 97 of these showing signs of diarrhea. We were still unable to detect either of these diarrheal-causing parasites in the diarrheal samples by repeated testing. Although these children were ruled out for diarrhea caused by these two parasites, other intestinal pathogens (bacteria, viruses, and other parasites) could still be responsible for their diarrheal symptoms (Hale et al., 2012; Conrad et al., 2017). Surveys of human *Cryptosporidium* infection conducted in several provinces in China (Guangxi, Guangdong, Henan, Hubei, Jiangsu, Shanghai, Yunnan, Xinjiang and Inner Mongolia) revealed prevalence rates ranging from 0.0 % to 16.5 % (Yang et al., 2021; Xu et al., 2020; Liu et al., 2024; Liu et al., 2020). The prevalence of *Cryptosporidium* infection varies significantly across different countries. For instance, the prevalence rates in India, Egypt, Cambodia, Ghana, Colombia, and Canada were 64.2 %, 1.4 %, 7.7 %, 5.2 %, 24.5 % and 15.7 %, respectively (Panchal et al., 2022; Naguib et al., 2018; Moore et al., 2016; Iqbal et al., 2015; Eibach et al., 2015). Furthermore, *G. duodenalis* infection has been reported in humans in up to 55 countries worldwide, with prevalence rate ranging from 0.03 % to 82.05 % (Sun et al., 2023). In developing countries, the prevalence of *G. duodenalis* infection is generally higher, particularly among children, with infection rate reaching 41 % in Ethiopia, 60.1 % in Rwanda, and 87 % in Uganda (Alemu et al., 2023; Ignatius et al., 2012; Al-Shehri et al., 2019). The prevalence of *G. duodenalis* infection among children in China, specifically in Zhejiang, Henan, Hubei, Anhui, and Shanghai, was found to be 0.9 %, 0.61 %, 1.4 %, and 2.4 %, respectively (Yu et al., 2019; Wang et al., 2017; Zhao et al., 2024). The prevalence of *Cryptosporidium* and *G. duodenalis* may be influenced by weather and climate conditions, sanitation infrastructure, access to clean water, with low temperatures and a dry climate potentially reducing their transmission chances (Wang et al., 2018; Geng et al., 2023). However, no *Cryptosporidium* and *G. duodenalis* were detected in human samples, which may be attributed to the following factors: first, as a coastal and mountainous region, Fujian Province benefits from concentrated residential areas and well-developed water purification and wastewater treatment facilities, effectively mitigating the risk of human infected with the two parasites. Secondly, major tourist cities including Fuzhou, Xiamen, Putian, Zhangzhou, Quanzhou, and Ningde boast a developed economy, complete infrastructure, and residents with strong health awareness, leading to a substantial reduction in the incidence of parasite infections caused by environmental factors. But Longyan, Sanming, and Nanping with relatively underdeveloped economies are still equipped with access to clean drinking water and sound medical services. Finally, although the samples cover most age groups, *Cryptosporidium* and *G. duodenalis* are more prone to infecting children under two years of age (Menu et al., 2022; Certad et al., 2017), with the relative scarcity of fecal samples from this age group in the present study also potentially accounting for the failure to detect these two pathogens. In this study, although we did not detect the two parasites in the human fecal samples, the large number of diarrheal samples underscores the need for ongoing efforts to enhance the detection of parasites and other potential pathogens. This is crucial for better understanding the causes of diarrheal diseases and reducing their impact on human health.

*Cryptosporidium* infections are common in dogs, with an overall global prevalence <10 % (Barbosa et al., 2023). A recent systematic review of the prevalence of *Cryptosporidium* in dogs reported that an overall mean prevalence was 7 %. The mean prevalence of *Cryptosporidium* detected by microscopy, coproantigens and molecular tools were 8.0 %, 7.0 %, and 6.0 %, respectively (Taghipour et al., 2020). In this study, among the 506 dog fecal samples collected in seven districts from Fujian Province, six samples tested positive for *Cryptosporidium*, with a prevalence rate of 1.2 % (6/506). Only one species of *Cryptosporidium* was identified, namely *C. canis*. *C. canis* infection is relatively common in dogs, although its prevalence varies significantly across regions. The prevalence rates of *Cryptosporidium* in Henan, Shanghai, Anhui, Beijing Guangzhou, and Yunnan were 3.77 %, 8.04 %, 1.58 %, 4.12 %, 2.92 %, 4.58 % respectively (Zhao et al., 2024). However, *C. canis* has been identified as a zoonotic species and has been found in human feces in several countries (Guo et al., 2021). Compared to other provinces in China, the prevalence of *Cryptosporidium* infection in dogs in Fujian Province is relatively low. The low infection rate is primarily ascribed to two factors: on the one hand, limitations in sample collection led to a small number of canine fecal samples from each region; on the other hand, all samples were obtained from urban areas (rather than rural or suburban areas), where breeders have a stronger awareness of rearing protection, with regular deworming and standardized management thus reducing the infection risk in companion animals. However, this does not rule out the potential risk of widespread infection within the local dog population. We observed the highest rates of *Cryptosporidium* infection in fecal samples collected from dogs in breeding kennels. This can be attributed to several factors: breeding kennels typically house mixed-breed dogs, many of the dogs are puppies/young with immature immune systems; the complex environment within breeding kennels; the high mobility of personnel, and the lack of strict isolation protocols for new dogs, all of which contribute to an increased risk of infection and cross-infection. The high prevalence of *Cryptosporidium* infection in breeding kennels aligns with the findings of previous research (Itoh et al., 2019; Giangaspero et al., 2006; Lupo et al., 2008). Notably, all samples tested positive for *Cryptosporidium* were obtained from dogs ≤1 year. Previous reports suggest that young age, the presence of diarrhea, and living in pet shops or shelters are risk factors associated with *Cryptosporidium* infection in cats and dogs (Barbosa et al., 2023). Statistical analyses revealed no significant difference in *Cryptosporidium* infection rates between male and female dogs, aligning with findings from Guangdong province (Li et al., 2021). *Cryptosporidium* infection in dogs has become an important public health issue as the proportion of dog-owning households in China increases. *C. canis* can infect both humans and dogs, and considering the close contact dogs with humans, they have the potential to be hosts for human cryptosporidiosis. Although studies have shown that dogs typically shed a lower number of *Cryptosporidium* oocysts, it is still to found 0–5000 oocysts per gram of feces in dog (Barbosa et al., 2023; Li et al., 2021). These oocysts have also been identified as contaminants in various types of food, particularly on numerous fresh vegetables and fruits, with at least 26 foodborne outbreaks reported worldwide (Ryan et al., 2018). Additionally, there are multiple reports about humans and animals in the same household being infected with *C. canis* due to their close contact with companion animals (Xiao et al., 2007; Khalifa et al., 2023). These findings highlight the importance of monitoring and controlling *Cryptosporidium* infections in both animals and humans to prevent zoonotic transmission and ensure public health safety.

*G. duodenalis* has been classified as a zoonotic parasitic disease affecting human health by the World Health Organization (WHO) since 1979 (WHO, 1979). Global attention has since been directed toward the prevention and control of *G. duodenalis*, leading to a steady decline in *G. duodenalis* infections over the years. The prevalence of *G. duodenalis* in dogs (1–57.9 %) varies widely and is usually <10 % (Barbosa et al., 2023). A meta-analysis estimated the global prevalence of *G. duodenalis* in dogs to be 2.61 % (112,513/4,309,451) (Bouzid et al., 2015). In this study, a total of 506 dog fecal samples from Fujian Province were tested for *G. duodenalis*, and only two samples tested positive, resulting in a prevalence rate of 0.40 % (2/506). This prevalence rate is lower than those reported in other regions of China, such as Liaoning (13.2 %, 27/205) (Li et al., 2013), Guangdong (9.7 %, 21/216) (Zheng et al., 2014), Sichuan (11.32 %, 18/159) (Zhang et al., 2017), and Yunnan (13.7 %, 36/262) (Wang et al., 2021). Several factors influence the prevalence of *G. duodenalis*, including age, the presence of diarrhea, socioeconomic status, the origin of the sample, and the use of anthelmintics (Barbosa et al., 2023). In this study, all *G. duodenalis* positive samples were from dogs ≤1 year old with diarrhea. This finding is consistent with previous reports suggesting that young and diarrheic dogs are more susceptible to *G. duodenalis* infection (Bouzid et al., 2015). With the development of social economy and the popularization of pet medical services, urban pet owners cover young (18–44 years), middle-aged (45–59 years), and old (≥60 years) groups. These owners generally regard dogs as family members, and are willing to invest more money in them and pay great attention to the health of companion animals, while also providing better living environments for companion animals, which collectively contribute to the low positive rate of *G. duodenalis*. Notably, we identified two assemblages of *G. duodenalis*, namely C and D, which are considered the most common *G. duodenalis* assemblages in dogs (Sun et al., 2023; Zhao et al., 2022). Previous reports have shown that assemblages C and D were similarly detected in samples from various locations across China, including three regions (Guangdong, Sichuan, and Shanghai) as well as from different sources (domestic dogs, stray dogs, and breeding kennels) (Sun et al., 2023). This suggests that assemblages C and D are prevalent throughout the Chinese region. However, *G. duodenalis* assemblages C, D, and F have been reported in humans and are considered to be assemblages with zoonotic potential (Jeske et al., 2022; Candela et al., 2021). As with *Cryptosporidium*, several studies have suggested possible transmission of *G. duodenalis* from pets to owners, particularly in socially-deprived areas (Barbosa et al., 2023). These findings highlight the zoonotic nature of *G. duodenalis*, emphasizing the need for improved hygiene practices, especially in households with pets, as well as in public spaces where dogs are commonly found. The high prevalence of the parasite in dog fecal samples suggests a potential risk of infection for both animals and humans, particularly for children, according to a recent study (Almeida et al., 2024). Studies have shown that dogs are not only potential carriers but may also play a significant role in the environmental contamination of *G. duodenalis* oocysts (Adam, 2001; Smith et al., 2020). Given the close human-animal relationship, particularly in households with young children and immunocompromised individuals, there is an increasing need for public health prevention measures targeting zoonotic transmission. Additionally, investigating dog fecal samples provides insight into potential zoonotic transmission routes, which is essential for formulating effective public health strategies in the region.

## 5 Conclusions

To our knowledge, this is the first report on the prevalence of *Cryptosporidium* and *G. duodenalis* in humans and dogs in Fujian Province, China. The present study describes the prevalence of *Cryptosporidium* in humans and dogs, as well as the combination of *G. duodenalis* in nine areas of Fujian Province. Although no positive results were detected in human samples, *C. canis* and *G. duodenalis* (assemblages C and D) were found in dog samples, which are capable of infecting humans. These findings not only expand our understanding of the distribution and genetic diversity of *Cryptosporidium* and *G. duodenalis* in China, but also provide valuable information for controlling intestinal parasite infections in dogs within Fujian Province. This study also has certain limitations, the number of samples from each region is relatively small, which cannot fully reflect the infection status of the two pathogens in the region; secondly, the sample collection was mainly from urban areas, lacking information supplementation from rural and suburban areas. Future studies should enrich samples from rural and suburban areas in various regions and collect a wider range of samples to increase the total sample size at each study site. In conclusion, the identification of these *Cryptosporidium* and *G. duodenalis* assemblages in dogs indicates a potential public health threat, particularly in regions where humans are in frequent close contact with infected animals. Consequently, these findings underscore the urgent need for public health education and the implementation of preventive measures. Strengthening preventive strategies and improving community hygiene practices will play a key role in mitigating the spread of these pathogens.

## CRediT authorship contribution statement

**Si-Ang Li:** Writing – original draft, Validation, Methodology, Investigation. **Yu-Ling Lin:** Validation, Methodology. **Yun-Peng Bai:** Validation, Methodology, Investigation. **Fuli Wen:** Visualization, Resources, Methodology. **Li-Yuan Huang:** Supervision, Methodology, Investigation. **Wen-Yuan Miao:** Supervision, Project administration, Methodology, Investigation. **Dong-Hui Zhou:** Visualization, Supervision, Project administration, Funding acquisition, Formal analysis, Data curation, Conceptualization.

## Ethics statement

The study was approved by the ethics committee of Fujian Agriculture and Forestry University (PZCASFAFU21032).

## Declaration of competing interest

The authors declare no conflict of interest.

## Data Availability

No datasets were generated or analyzed during the current study.
